# Value of DSA in the Diagnostic Workup of Pulsatile Tinnitus

**DOI:** 10.1371/journal.pone.0117814

**Published:** 2015-02-17

**Authors:** Cornelius Deuschl, Sophia Göricke, Carolin Gramsch, Neriman Özkan, Götz Lehnerdt, Oliver Kastrup, Adrian Ringelstein, Isabel Wanke, Michael Forsting, Marc Schlamann

**Affiliations:** 1 Institute of Diagnostic and Interventional Radiology and Neuroradiology, University Hospital of Essen, Essen, Germany; 2 Clinic of Neurosurgery, University Hospital of Essen, Essen, Germany; 3 Clinic of Otolaryngology, University Hospital of Essen, Essen, Germany; 4 Clinic of Neurology, University Hospital of Essen, Essen, Germany; Heinrich-Heine University, GERMANY

## Abstract

**Objectives:**

Pulsatile tinnitus (PT) is a rare complaint, but can be a symptom of life-threatening disease. It is often caused by vascular pathologies, e.g. dural arteriovenous fistula (dAVF), arteriovenous malformation (AVM) or vascularized tumors. The current diagnostic pathway includes clinical examination, cranial MRI and additional DSA. The aim of this study was to evaluate the diagnostic impact of DSA in the diagnostic workup of patients with PT in comparison to MRI alone.

**Methods:**

Retrospectively, 54 consecutive patients with pulsatile tinnitus were evaluated. All patients had a diagnostic workup including cranial MRI and DSA. MRI examinations were blinded to the results of DSA and retrospectively analyzed in consensus by two experienced neuroradiologists. The MR-examinations were evaluated for each performed sequence separately: time-of-flight-angiography, ce-MRA, T2, ce-T1-sequence and ce-T1-sequence with fat saturation.

**Results:**

37 of the 54 patients revealed a pathology explaining PT on MRI, which was detected by the readers in 100% and proofed by means of DSA. 24 dAVF, four paraganglioma, two AVM and seven more pathologies were described. All patients without pathology on MRI did also not show any pathology in DSA.

**Conclusions:**

MR imaging is sufficient to exclude pathology in patients with pulsatile tinnitus.

## Introduction

Pulsatile tinnitus (PT) is a rare symptom potentially provoked by a variety of pathologies and can be sign of life-threatening disease [1,2,3]. It is characterized by a noise synchronous to the heartbeat, which is transmitted to the inner ear and generated by non-laminar blood flow. PT can be classified into `objective´ when detecTable with auscultation or `subjective´ when only the patient perceives the noise. The etiology of PT in general can be vascular, nonvascular or remain cryptogenic without radiologically identifiable cause [4]. The vascular genesis of PT can be separated into arterial or venous pathologies. Frequent vascular pathologies of PT are dural arteriovenous fistula (dAVF), arteriovenous malformations (AVM), sigmoid sinus diverticulum, carotid-cavernous fistulae or ICA stenosis [5,6]. Frequent nonvascular causes of PT are paraganglioma, intracranial hypertension or systemic disorders e.g. anemia [1,7,8].

About 25–30% of patients with PT have no imaging findings explaining the symptom [9,10]. Potential life-threatening diseases like dAVF need to be detected and—depending on their classification—treated due to the risk of bleeding [11,12,13]. DAVF Type III-V classified by Cognard/Merland have a significantly higher risk of bleeding and usually therapy is recommended [12]. In patients with dAVF, PT is described in 41% of patients not presenting with intracranial hemorrhage [14].

There are different imaging strategies due to the numerous possible causes of PT. The current diagnostic pathway includes a detailed neurological and otolaryngeal examination and cranial MRI (cMRI). After that frequently diagnostic digital subtraction angiography (DSA) is recommended, which is still considered as gold standard in evaluation vascular pathologies [10,15].

The aim of this retrospective study was to evaluate the diagnostic impact of DSA in the diagnostic workup of patients with PT in comparison to MRI alone.

## Materials and Methods

The DSA database was evaluated for patients with PT presenting in our department between 2003–2013. All patients with both, DSA and MRI examinations were included in this study. Patient records were anonymized and de-identified prior to analysis. Due to the retrospective character of our study consent of participants was not given. The study was approved by the ethics committee of the University Duisburg-Essen.

Overall 54 consecutive patients (37 women; 17 men; mean age 51.5 years; range 18–73 years) with PT were included in this study. All patients had a diagnostic workup with MRI and DSA. MRI and DSA were performed on average within a time span of 31 days (median of 21 days). All patients suffered from PT both on the day of MRI examination and DSA. Two experienced neuroradiologists (certified for >10 years) reanalyzed MRI in consensus. The readers were informed of the lateralization of PT, but were blinded for DSA results and initial MRI report. Due to the different MRI protocols we evaluated each MRI sequence separately. The following sequences were analyzed: time-of-flight angiography (TOF-angio), ce-MRA, T2, ce-T1 and ce-T1-fs. All sequences covered the entire brain. The ce-MRA covered additionally the supraaortal vessels.

Each sequence was analyzed separately for its diagnostic value on a two point scale (pathologic or non- pathologic). MRI examinations were performed on several different MRI-scanners (Siemens Medical Healthcare, Erlangen Germany: Symphony (1.5T): 13 patients; Espree (1.5T): four patients; Avanto (1.5T): three patients; Sonata (1.5T): two patients; Aera (1.5T): two patients; Skyra (3T): one patient; Biograph_mM (3T): one patient and 28 external MRIs. The external MRI examinations were performed on a variety of different MRI scanners; 24 of the examinations were performed at 1,5T and four at 3T. Due to the retrospective character of the study and the different MRI-scanners and scan protocols we do not present a detailed protocol of all sequence-parameters.

## Results

### Diagnosis made by DSA

**Fig 1:**
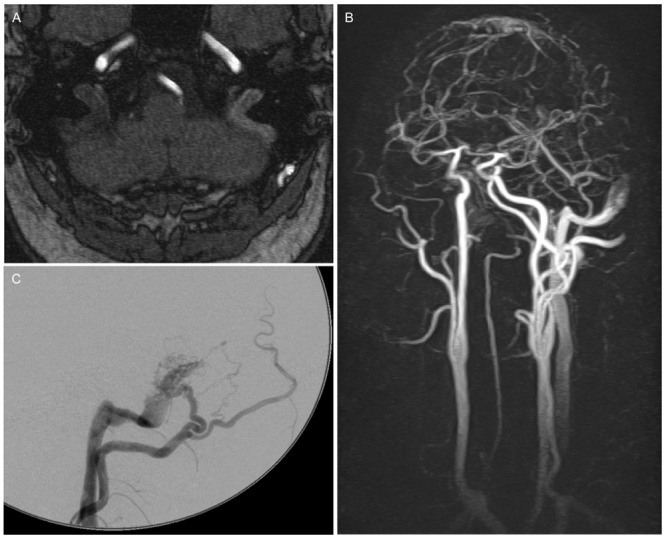
46 year old male patient with left sided PT since 6 months. TOF (A), ce-MRA (B) and DSA (C): dAVF I by Cognard/Merland classification of the sigmoid sinus.

**Fig 2:**
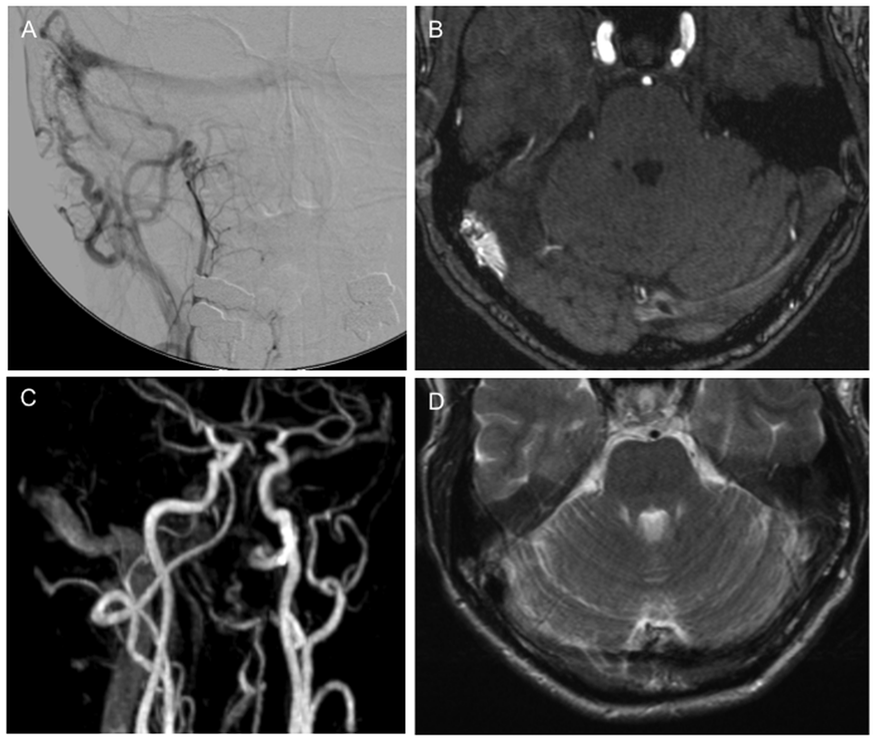
53 year old patient with right-sided pulsatile tinnitus and severe headache since 4 months. DSA (A), TOF (B), ce-MRA (C) and T2 (D): DAVF IIa by Cognard/Merland classification at the sigmoid/transverse sinus.

**Fig 3:**
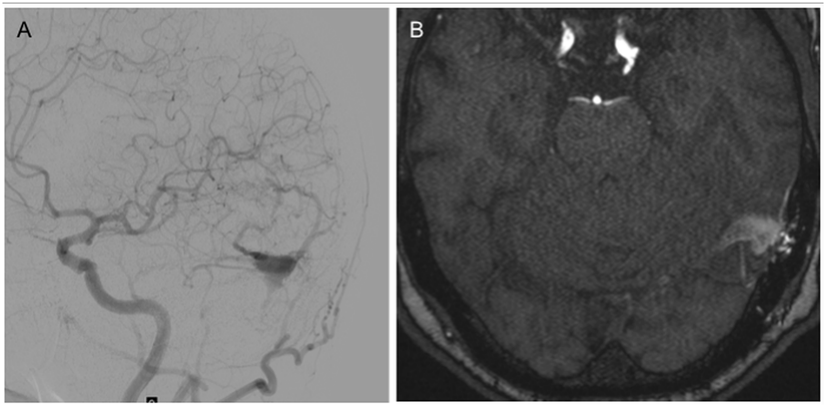
64 year old male patient with PT on the left ear since 4 months. DSA of the left carotid artery shows a left sided dAVF Type III Cognard/Merland classification (A). The venous drainage leads direct into a trapped left sided transverse sinus into a cortical vein, which is not dilatated (B).

**Fig 4:**
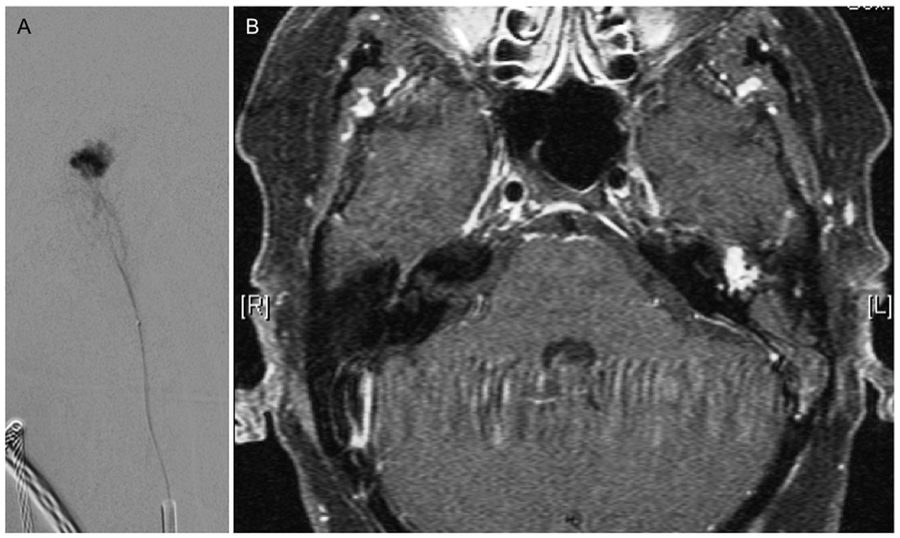
42 year old patient with left sided PT for one year. A: Superselective angiographic microcatheter injection before preoperative embolization with particles. B: MRI ce-T1 fs shows a Glomus tympanicum paraganglioma: enhancing mass in loco typico.

Fifty-four consecutive patients were included. Final diagnosis was made by means of DSA, which is considered to be the gold standard in visualization intracranial vessels. PT-causing pathology was detected in 37 of 54 patients by DSA (Table 1). In total 24 patients had dAVF (Figs. 1–3) and four patients had a paraganglioma (Fig. 4). Two patients with AVM, three patients with extracranial fistula, two patients with carotid-cavernous fistulae, one patient with stenosis of the internal carotid artery and one patient with an aneurysm of the internal carotid artery were detected. In 17 patients no pathology was found.

**Table 1:** Patient group with PT according to final diagnosis based on DSA and MRI.

Pathology	Diagnosis made with DSA n (%)	Diagnosis made with MRI n (%)
dAVF	24 (44%)	24(44%)
Paraganglioma	4 (7%)	4 (7%)
Cavernosus fistula	2 (4%)	2 (4%)
AV malformations	2 (4%)	2 (4%)
Extracranial AV fistula	3 (6%)	3 (6%)
ACI stenosis	1 (2%)	1(2%)
Aneurysm	1 (2%)	1 (2%)
No pathology	17 (31%)	17 (31%)

### Diagnosis made by MRI

Tinnitus-causing pathology was seen in 37 of 54 patients in MRI (Table 1). The most frequent cause for PT was dAVF (24 patients). The second most frequent cause was paraganglioma in 4 patients. Two patients had an AVM supposed to be the cause for PT. Carotid-cavernous fistulae were seen in two patients. In one patient an aneurysm of the internal carotid artery was the reason for PT, which vanished after coil embolisation. Stenosis of the internal carotid artery was the only detecTable reason of PT in one patient. Extracranial arteriovenous fistulae were found in three patients (Table 1). No morphological correlate for PT was identified on MRI of 17 patients and DSA did not reveal any further pathology in these patients.

### MRI sequences

To allow the comparison of the diagnostic impact of the different MRI sequences, the patients with pathological findings in DSA were evaluated separately (n = 37) (Table 2).

**Table 2:** Patient group with pathological correlate to PT with TOF-, ce-MRA-, T2-, ce-T1 and ce-T1-fs-sequences.

MRI-sequence	Patients with pathology	Pathologic	Non pathologic	Sensitivity
TOF	26	25	1	96%
Ce-MRA	20	19	1	95%
T2	34	20	14	59%
Ce-T1	23	17	6	74%
Ce-T1-fs	8	4	4	50%

Of the 37 patients with pathologic findings on DSA 26 had received TOF angiography. In 25 of those patients TOF angiography was suspicious for pathology, corresponding to a sensitivity of 96% (Table 2). In one patient no pathology was detected in TOF angiography, but in DSA. This pathology was detected in ce-MRA (Table 2). However one patient with no pathologic finding in DSA showed a suspicious flow artifact in TOF angiography. Out of the 37 patients with pathology 20 patients received ce-MRA, of which 19 showed pathologic findings (sensitivity of 95%) (Table 2). No correlate for PT was found in 6 of 23 patients in ce-T1 (sensitivity of 74%) (Table 2). Sensitivity was 59% for T2 and 50% for ce-T1-fs (Table 2). All patients with an explaining pathology for PT in DSA had at least one MRI-sequence suspicious for pathology. MRI showed the PT explaining pathology in all cases.

Details of the tinnitus characteristics were documented in 20 patients of whom14 had a subjective PT (5 dAVF, 1 AVM, 2 cavernosus fistulae and 6 patients without pathology) and 6 patients an objective PT (5 dAVF and 1 traumatic AV fistula of the vertebral artery). All patients with objective PT had morphological correlate which was detecTable on DSA as well as on MRI.

All dAVF patients (n = 24) were graded according to the Cognard/Merland classification in DSA [12].

## Discussion

This is the first systematic study evaluating the diagnostic value of MR-imaging compared to DSA in PT. MRI alone was sufficient for diagnostic purposes in patients with PT in all cases. DSA revealed no further information in patients without MRI pathology. This is relevant as DSA is generally considered necessary if MR-imaging in PT patients is negative [4,16].

DAVF is the most common potentially life-threatening reason for PT. DSA is still considered as gold standard for grading of av-fistulae, but if MRI/MRA reveals no pathology, dAVF can be excluded according to our data.

The MRI-protocol should contain TOF-angiography for detecting intracranial stenosis, aneurysm and fistula.

Ce-MRA was already described as an important tool in diagnosing lesions responsible for PT [15]. It is sensitive for screening supraaortal vessels for av-fistula and stenosis (Figs. 1 and 2) [17]. In our clinical experience ce-MRA is also sufficient in identifying sigmoid sinus diverticulum, especially when the source data, which are usually acquired in coronal orientation, is reformatted in transversal orientation.

Ce-T1 fs and T2 in transversal orientation give a good anatomical overview and can give hint for fistula due to enlarged vessels. The slice thickness should not exceed 5 mm. Additionally ce-T1 with spectral fat saturation of the skull base should be performed to exclude neoplasms like paraganglioma. The slice thickness should not exceed 3 mm, 2 mm is preferable.

Recently susceptibility weighted imaging was described to be sensitive for detecting dAVF, so that we additionally would recommend to use SWI as well [18,19,20]. Potentially acquired SWI images were not evaluated in this study, because of the limited availability in our collective (n = 6). Arterial spin labeling is another technique that is helpful to identify shunting lesions [21]. Also TWIST angiography is a new promising technique that is very helpful in daily routine [4,16,22,23] Due to the retrospective character of our study and the limited availability of these sequences in the past we did not separately evaluate these sequences. Future larger studies need to evaluate the diagnostic impact of these sequences.

In our collective most examinations (n = 48) were performed on 1.5T MRI scanners and only a few on 3T MRI scanners (n = 6). Within this retrospective study with different MRI scanners and different sequence protocols in a time period from 2002 to 2013 all pathologies were detected by MRI with a sensitivity of 100%. As it is to be expected that 3T images in total will not be less sensitive than 1.5T images, we generally recommend examinations at field strength of at least 1.5T.

Our study has some limitations. Due to the retrospective character, different MRI scanners as well as different MRI protocols were used. However this is more or less a “worst-case-scenario”. If, even under these heterogenous conditions the sensitivity of MRI is excellent, the diagnostic value of specific dedicated MR protocols will be even better.

Moreover high-resolution computed-tomography angiography is an important tool to exclude rare cases like carotid-cochlear dehiscence, aberrant carotid-artery or a persistent stapedial artery [9,10]. We think that CT/CTA is an additional modality, which should be used in patients with unsuspicious MRI.

Our recommended examination protocol is a synopsis of the evaluated examinations (Table 2). Aim of our protocol is to detect vascular pathology of the supraaortal and intracranial vessels (e.g. AVF, AVM and stenosis) and to exclude tumor. However despite these heterogeneous MR protocols experienced neuroradiologists were able to identify all vascular pathologies in our patient group.

The series contained a high number of dAVF (n = 24/44%). A meta-analysis performed by Hofmann (2013) describes a relative frequency of dAVF in patients with PT of about 7% [9]. This high percentage of patients with dAVF in our collective is caused by the fact of being a center for treatment av- fistulae. DAVFs are the most common and potentially life threatening cause for PT. According to our results it is possible to rule out the diagnosis of an av-fistulae by means of MRI.

Patients with PT need treatment in case dAVF (IIb-V) [12]. Moreover our study population is different to general population of PT patients because pathologies which do not require DSA are excluded, e.g. idiopathic intracranial hypertension or systemic causes. The inclusion criteria of our study demanded the presence of MRI and DSA. In the assumed case of a patient with negative MRI/MRA and high psychological burden caused by PT we discus the situation with the patient and clarify that we expect no pathological finding in DSA due to the results of this study.

All patients with objective PT within our study had pathological findings in MRI/MRA, confirmed by previous publications describing a high correlation of objective PT and pathology [16].

**Fig 5:**
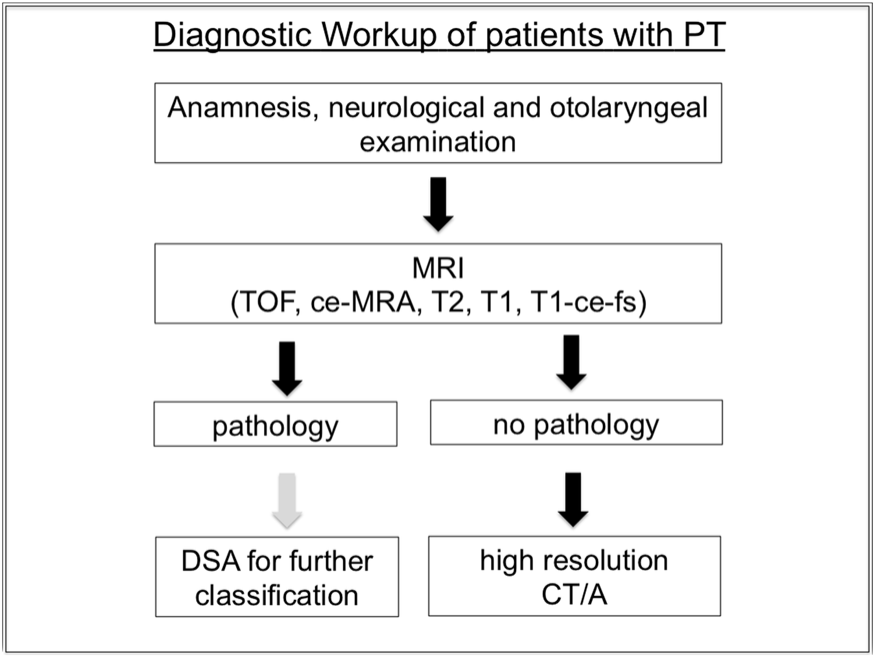
Diagnostic Work-Up for patients with PT. DSA is only recommended in patients with a finding of a vascular pathology in MRI.

According to our findings DSA is only recommended in patients with PT when MRI is suspicious for vascular pathology like dAVF (Fig. 5), where DSA still has an impact on treatment planning. However, as mentioned before, if all patients with PT would receive a dedicated and high end MR examination DSA could probably be totally replaced.

If MRI would one day be able to detect the venous drainage pattern properly for classification of dAVF it might be able to replace DSA even in patients with fistula.
